# Retraction: Modification of Glycosylation Mediates the Invasive Properties of Murine Hepatocarcinoma Cell Lines to Lymph Nodes

**DOI:** 10.1371/journal.pone.0222417

**Published:** 2019-09-06

**Authors:** The PLOS ONE Editors

After publication of this article [1], the following concerns were raised about the published figures:

- The FUT8 panel in Fig 2B appears similar to the ST6Gal1 panel in Fig 6A when horizontally flipped;
- There are similarities between cell staining patterns in the following panels of Fig 4B and 4C in [1] and the indicated panels of Fig 3A and 3B in [2]:
  ○ 0 μg/ml panel of Fig 4B [1] and 16h panel of Fig 3B [2];
  ○ 5 μg/ml panel of Fig 4B [1] and 20 μg/ml panel of Fig 3A [2];
  ○ 0 hr panel of Fig 4C [1] and 8h panel of Fig 3B [2];
- Several stained cells in Fig 4B and 4C appear to have highly similar morphologies and staining patterns;
- Areas within Fig 4B (1 and 10 μg/ml panels) of [1] are similar to data shown in Fig 2D and 2E of [3];
- An area within the 0 μg/ml panel of Fig 4B [1] appears similar to data shown in the Hca-F and control siRNA panels of Fig 3A in [4]; the upper portion of the 5 μg/ml panel in Fig 4B [1] is similar to a region of the COL8A1 siRNA panel in Fig 3A of [4];
- There are clusters of stained cells that appear to be duplicated within and across panels of Fig 5B and 6B in [1]. Also, within the first panel of Fig 6B the left portion of the panel appears to be duplicated and flipped vertically in the central portion of the panel.

We raised these concerns to the authors, who apologized and provided supporting image files as well as replication data for the results in question. However, the image data provided did not resolve the published image concerns. Therefore, the *PLOS ONE* Editors retract this article due to concerns about the integrity, validity, and reliability of the published results.

All authors agreed with the retraction.

Note: Permissions were not obtained to use the images described above in the second, fourth, and fifth bullet points, in Fig 4B and 4C of this *PLOS ONE* article [1], and so those images are excluded from the article’s CC-BY license. See [2–4] for information about the licenses that apply to this material.
